# Staying Physically Active Is Associated with Better Mental Health and Sleep Health Outcomes during the Initial Period of COVID-19 Induced Nation-Wide Lockdown in Jordan

**DOI:** 10.3390/ijerph19020776

**Published:** 2022-01-11

**Authors:** Yazan A. Al-Ajlouni, Su Hyun Park, Jude Alawa, Ban Dodin, Ghaith Shamaileh, Nour Makarem, Katherine M. Keyes, Dustin T. Duncan

**Affiliations:** 1 Department of Epidemiology, Columbia University Mailman School of Public Health, New York, NY 10032, USA; suhyun.park@nus.edu.sg (S.H.P.); nm2968@cumc.columbia.edu (N.M.); kmk2104@cumc.columbia.edu (K.M.K.); dd3018@cumc.columbia.edu (D.T.D.); 2 School of Medicine, New York Medical College, Valhalla, NY 10595, USA; 3 School of Medicine, Stanford University, Stanford, CA 94305, USA; jude.alawa@gmail.com; 4 School of Medicine and Public Health, University of Wisconsin, Madison, WI 53705, USA; bandodin@gmail.com; 5 Department of Cell and Molecular Biology, Tulane University School of Science and Engineering, New Orleans, LA 70118, USA; gshamaileh@tulane.edu

**Keywords:** COVID-19, lockdown, physical activity, mental health, sleep health

## Abstract

Jordan, a Middle Eastern country, initially responded to an outbreak of COVID-19 cases within its own borders by imposing a 7-week strict lockdown and closure of international and domestic travel. Such measures drastically influenced lifestyle behaviors of the population. This study aimed to investigate the prevalence of physical activity, and its association with mental and sleep health outcomes among Jordanians during a period of COVID-19 induced lockdown. Validated questionnaires were administered using a web-based platform to evaluate moderate-to-vigorous physical activity (MVPA), anxiety and depressive symptoms, sleep health, and sociodemographic characteristics. A modified Poisson regression model with robust error variance was used to estimate adjusted prevalence ratios (aPRs) and 95% confidence intervals (CIs). Compared to participants who met the guidelines, those who did not had significantly higher prevalence of moderate or severe anxiety symptoms than that of minimal or mild anxiety symptoms and increased depressive symptoms. Insufficient MVPA was associated with higher prevalence of poor sleep quality, short sleep duration (<7 h) and sleep problems. Overall, sufficient MVPA was associated with better mental and sleep health during the COVID-19 induced nation-wide lockdown in Jordan. While further research is necessary, promoting physical activity during the lockdown could potentially improve mental and sleep health outcomes among the population.

## 1. Introduction

In December 2019, an epidemic of cases associated with acute lower respiratory infections was detected in Wuhan, China. These cases were caused by a novel coronavirus called severe acute respiratory syndrome coronavirus 2 (SARS-CoV-2). In the next several weeks, the coronavirus disease 2019 (COVID-19) had spread across China and several other countries across the world [1]. In March 2020, the World Health Organization (WHO) declared the COVID-19 outbreak to be a global pandemic and a public health emergency requiring immediate international concern [2]. Jordan, an Arab country in Western Asia, reported its first case of COVID-19 on 2 March 2020 [3]. In response to the rapid spread of the virus in countries across the globe, Jordan imposed several national restrictions to prevent any further spread of the virus within its borders. On 14 March, the Jordanian Government issued a travel ban blocking entry to or departure from any of the country’s borders. A nation-wide lockdown was imposed on 17 March 2020 to restrict the movement of residents in public spaces [4] and allow physical distancing. During the nation-wide lockdown, movement outside residences was limited to certain days of the week, and all public spaces, including parks and recreational spaces, were shut down. In addition, groceries were only made available through delivery services, and driving was prohibited throughout the country. These restrictions lasted nearly two months until 3 May 2020.

Though imposing such restrictions has been reported to be effective in containing the spread of COVID-19 [5,6], the nation-wide lockdown and quarantine is expected to have perpetuated social isolation, changes in social habits, and disruptions to lifestyle routines (e.g., decreased socialization and involvement in group sports). In turn, this may lead to adverse behavioral health outcomes (e.g., physical inactivity and poor sleep) and mental health (e.g., stress, anxiety) [7,8,9,10,11]. Previous research demonstrates that epidemics and large-scale disasters (e.g., natural, environmental) negatively impact mental and behavioral health, leading to posttraumatic stress disorder, depression, anxiety, substance use disorders, domestic violence, child abuse, and poor sleep health, among other conditions [12,13]. A study found that 36.8% of 932 UK adults reported poor mental health as a result of social isolation stemming from COVID-19 [14]. Furthermore, a recent study identified an increased prevalence of anxiety, depressive symptoms, and poor sleep health among Jordanian adults during the COVID-19 imposed nationwide lockdown in Jordan, in comparison to pre-lockdown prevalence reports in the literature [15]. Sleep and the circadian system cooperate to organize and regulate effective immune responses. Given their role in mediating an immune response that can aid in combating the COVID-19′s highly contagious nature and varied pathophysiology [16,17], this relationship reinforces the importance of monitoring mental health and sleep health outcomes in the context of COVID-19 pandemic [17,18,19,20,21,22]. As such, it is vital to investigate behaviors that may affect mental and sleep health, especially in contexts of lockdowns, due to their role in mediating immune responses.

Regular physical activity may play a role in maintaining good mental and sleep health during the COVID-19 pandemic. The 2020 WHO guidelines on physical activity and sedentary behavior recommend that all adults undertake 150–300 min of moderate-intensity, or 75–150 min of vigorous-intensity physical activity, per week [23]. Evidence in non-pandemic contexts shows that sustained physical activity is associated with improved sleep duration and quality, mental health, and cardiovascular, respiratory and immune functions [24,25,26,27,28,29,30]. Recent meta-analyses also show that physical activity can protect against symptoms of depression or anxiety, regardless of age or geographic location [31,32]. Nevertheless, evaluations of these associations during COVID-19 remain in their infancy. Research from Italy, Spain, the United Kingdom, and cross-national assessments report that reductions in moderate-to-vigorous physical activity (MVPA) as a result of lockdown measures to combat COVID-19 are associated with negative psychosocial health outcomes (e.g., mental, emotional, social, and spiritual health) [1,33,34,35,36]. As of result of COVID-19 mobility restrictions, however, one study found that sleep duration actually increased, while physical activity decreased significantly [37]. Another study reported that physical activity and sleep quality only declined in physically active individuals, suggesting that there may be a differential association between sleep health and physical activity depending on an individual’s physical activity levels prior to the pandemic [38]. Despite the expected beneficial impact of physical activity on mental and sleep health during the lockdown, limited data during the pandemic exists to elucidate differential results. These associations and their nuances have yet to be sufficiently studied, especially within the Middle Eastern context, where comparatively less public health data are available, and the population faces unique stressors, socioeconomic factors, and chronic disease rates. It is important to understand these previously investigated associations in the context of the pandemic to identify population groups at higher risk of poor mental and sleep health and their possible impact on disease risk and health span.

Therefore, this study aimed to investigate the prevalence of physical activity, and its associations with mental and sleep health outcomes during the COVID-19 induced nation-wide lockdown in Jordan. To the best of our knowledge, this study is one of the earliest reports to evaluate associations of physical activity with mental and sleep health in Jordan, as well as the Middle East region, during the COVID-19 pandemic. We hypothesized that the nation-wide lockdown due to the pandemic would have a negative impact on engagement with physical activity and that sufficient MVPA would be positively associated with mental and sleep health outcomes among Jordanian adults, after controlling for sociodemographic covariates.

## 2. Methods

### 2.1. Study Design and Participants

Design and methods of this study were described in detail elsewhere [15]. Briefly, cross-sectional data were collected from participants who met the eligibility criteria of: (1) being a Jordanian citizen aged between 18 and 65 years; (2) and resided in Jordan for at least two full weeks at the time of the COVID-19 induced nation-wide lockdown, from 14 to 28 March 2020. Participants’ sociodemographic information and data on aspects of mental health and sleep health metrics were collected using Qualtrics (i.e., an online survey administration tool) [39]. The survey was developed in English and then translated into Arabic by three bilingual researchers independently. To ensure clarity and readability, the survey was reviewed by three independent English and Arabic speakers. A link to the survey was advertised on Facebook (i.e., a social media platform) for four consecutive days between 29 March and 1 April. In order to circumvent duplicate responses, the “prevent ballot box stuffing” option in Qualtrics (which kept people from taking the survey more than once) was employed, and a manual inspection of internal protocol (IP) addresses was performed for given responses. A total of 3462 people opened the survey link posted on Facebook and were directed to the survey. Of these, 2202 people met the eligibility criteria, wherein 962 were did not complete the survey fully. Out of the eligible participants, 1240 people completed the survey fully, reaching the completion rate of 56.4%. The survey took an average of seven minutes to complete. The University of Jordan Hospital Institutional Review Board approved the ethics, protocol, and procedure of this study.

### 2.2. Sociodemographic Characteristics

Sociodemographic characteristics were measured using four variables: age, gender, employment status and city of residence. Participants were asked to report their age in years; gender as either male or female; employment status as either employed, self-employed, unemployed, student or retried; and their city of residence within Jordan. Cities were categorized into 3 different groups based on region: (1) Northern Jordan, including Ajloun, Balqa’a, Irbid, Jerash, Mafraq, and Zarqa; (2) Central Jordan including Amman, Madaba, and Salt; and (3) Southern Jordan including Aqaba, Karak, Ma’an, Petra, and Tafila.

### 2.3. Physical Activity

Two items from the International Physical Activity Questionnaire (IPAQ) were used to measure the frequency (i.e., days per week) and duration (i.e., minutes per day) of MVPA over the past seven days. The IPAQ has been a well-utilized tool in health research and shown its validity and reliability across numerous populations [40,41,42]. The values of the two items were multiplied to calculate the number of minutes spent performing MVPA per week. Participants were then classified into two groups based on whether they met the 2020 Adult Physical Activity Guidelines of at least 150 min of MVPA per week outlined by The World Health Organization (WHO) [43,44,45].

### 2.4. Mental Health

#### 2.4.1. Anxiety Symptoms

Anxiety symptoms were measured using the General Anxiety Disorder 7-item anxiety (GAD-7) scale. Each item asked participants how often they were agitated by a given symptom during the last two weeks of March 2020, with responses being “not at all (score: 0)”, “several days (score: 1)”, “more than half the days (score: 2)” and “nearly every day (score: 3)”. Scores from the seven items were summed to calculate an overall score ranging from 0–21 such that higher scores were indicative of more severe anxiety symptoms. The score was categorized into four levels: minimal (score: 0–4), mild (5–9), moderate (10–14) and severe (15–21), and then participants were dichotomized as having minimal/mild versus moderate/severe anxiety. The items showed good internal consistency with a Cronbach’s alpha of 0.87.

#### 2.4.2. Depressive Symptoms

The Center for Epidemiologic Studies Depression Scale (CES-D; 10 items) was used to measure depressive symptoms. The scale was modified to include items that were deemed consistent with cultural and societal concerns as they relate to depression and that fully capture the denotation of these items when translated to Arabic. Modifications involved minor wording changes to be consistent with Arabic translation. No addition or removal of any entire novel items was performed. For each item, with a recall period of 7 days, response options were as follows “rarely or none of the time (<1 day)”, “some or a little of the time (1–2 days)”, “occasionally or a moderate amount of time (3–4 days)”, and “most or all of the time (5–7 days)”, which corresponded to the scores of 0, 1, 2, and 3, respectively. Three of the 10 items were reverse coded owning to their positive connotation, and a total summed score was calculated on a scale of 0–30. For our analyses, a binary variable was created based on the median (a score of 6) as a cutoff value. Good internal consistency was indicated with a Cronbach’s alpha of 0.82.

### 2.5. Sleep Health

Sleep health was measured using three items adopted from the Pittsburgh Sleep Quality Index (PSQI), a validated measurement for sleep quality [46]. The first item asked participants the following question: “During the past month, how would you rate your sleep quality overall?” [47]. Participants were asked to respond to the questions with “very good”, “fairly good”, “fairly bad” and “very bad”. The four response options were dichotomized as good sleep quality (i.e., “very good”, “fairly good”) and poor sleep quality (i.e., “fairly bad”, “very bad”) [48,49,50,51]. In the second item, the duration of sleep was measured by asking “During the past month, how many hours of sleep did you get each night? (which may be different from the number of hours you spent in bed)” [47]. Responses were recorded in hours as integers with one decimal place rounded to the nearest half (e.g., 7.2 would be coded as 7.0; 7.3 would be coded as 7.5). Participants with less than seven hours of sleep were classified as having a short sleep duration [48,52,53]. The third item gauged problems with sleep by asking participants if they had experienced any of the three sleep-related problems during the past two weeks of the lockdown in March 2020. The three statements were as follows:

1. “I had trouble sleeping because I could not get to sleep within 30 min” (i.e., problems falling asleep);
2. “I had trouble staying awake while driving, eating meals, or engaging in social activity” (i.e., problems staying awake during the day, which is also known as daytime sleepiness);
3. “I took medicine (prescribed or “over the counter”) to help me sleep”.

Participants were given “yes” or “no” responses to the aforementioned questions.

### 2.6. Statistical Analysis

Descriptive statistics including frequencies and percentages were performed for all variables. A modified Poisson regression model with robust error variance was conducted to assess associations of MVPA with mental and sleep health outcomes. The choice of modified Poisson regression model was made due to the high prevalence of mental and sleep health outcomes [54,55,56,57,58,59]. Adjusted prevalence ratios (aPR) and 95% confidence intervals (CI) were estimated. Demographic variables were included in the models as covariates. *p*-values were two-sided, and a statistically significance level of 0.05 was applied. All analyses were conducted using Stata 16.0 (StataCorp, College Station, TX, USA).

## 3. Results

Participants’ characteristics and their descriptive statistics are presented in Table 1. Over 60% of the participants were less than 40 years old, with the mean age of 37.4 (standard deviation (SD) = 11.0) years. More than half (52.9%) of the participants were males. Most participants were employed (54.3%), but under a quarter was unemployed (17.6%) or retired (4.6%). The majority (78.2%) lived in the central region of the country, followed by the northern regions (19.1%). Only 31.5% of the participants met the physical activity guidelines. Regarding levels of anxiety symptoms, the participants had mild (33.8%), moderate (12.9%) or severe symptoms (6.3%). Moreover, about one fourth of our sampled participants scored in each of the four quartiles for the measures of depressive symptoms: 21.5% in the highest quartile, 26.8% in the third quartile, 24.8% in the second quartile, and 26.9% in the lowest quartile. In terms of sleep health outcomes, 17.9% of the participants experienced poor sleep quality, 46.8% had shorter sleep durations (i.e., equal to or less than 7 h per night), and 63.7% had at least one sleep disturbance.

Results from modified Poisson regression models are presented in Table 2. Compared to participants who met the physical activity guidelines (e.g., ≥150 min of weekly MVPA), those who did not meet the physical activity guidelines (e.g., less than 150 min of weekly MVPA) had significantly higher prevalence of moderate or severe anxiety symptoms compared to minimal or mild anxiety symptoms (aPR = 1.36; 95% CI = 1.05–1.77), and increased levels of depressive symptoms (aPR = 1.30; 95% CI = 1.14–1.49). Furthermore, and when testing the relationship between physical activity and sleep health, participants who did not meet the guidelines for MVPA had higher prevalence of poor sleep quality (aPR = 1.68; 95% CI = 1.24–2.26), short sleep duration (aPR = 1.15; 95% CI = 1.00–1.31), and sleep problems (aPR = 1.22; 95% CI = 1.10–1.35).

## 4. Discussion

To the best of our knowledge, this study is the first to investigate the prevalence of physical activity, and its associations with mental and sleep health outcomes among the Jordanian population during the COVID-19 induced nation-wide lockdown. In our study, less than a third (31.5%) of the participants met the recommendations of 150 min or more of MVPA per week, meaning that most participants did not engage in MVPA. Furthermore, our findings suggested that sufficient MVPA (i.e., at least 150 min per week) was significantly associated with lower levels of depressive and anxiety symptoms, better sleep quality, longer sleep durations, and less problems during sleeping. These findings supported our hypothesis and were consistent with previous research conducted among various populations during periods of lockdowns. For instance, decreased levels of weekly physical activity were negatively associated with well-being in the French Reunion Island [60] and mood in England [61]. In the same way, Ernsten et al. reported that symptoms of anxiety and depression were considerably lower in physically active Norwegian adults [62]. Additionally, the findings of this study suggest that during lockdown, Jordanian adults may have engaged in drastically lower levels of physical activity. In a study conducted among Middle Eastern and North African countries, Jordanian adults had the highest pooled prevalence measures of sufficient physical activity levels, among both females and males (94.2% and 95.5%, respectively) [63]. However, it is important to note that due to differing definitions of physical activity, it is difficult to make precise comparisons with other reports in the literature that continue to be inconsistent. For instance, another study published by Walke et al., reported that 51.8% of Jordanian adults did not engage in moderate physical activity [64].

Moreover, our findings are consistent with studies demonstrating that physical activity may associated with more favorable sleep health. In the pre-pandemic context (i.e., no lockdown imposed), studies on the relation between physical activity and sleep health provided mixed results but are generally suggestive of a bidirectional relationship between these lifestyle behaviors. A 2015 review and meta-analysis of the existing literature showed that both acute and regular exercise had small to moderate beneficial effects on sleep duration, overall sleep quality, and aspects of sleep quality such as sleep efficiency and wake time after sleep onset [30]. Mechanisms underlying the relation between physical activity and sleep are not fully elucidated, but some of the hypothesized underlying pathways include improved emotional regulation, reduced anxiety and positive mood changes [65], changes in body temperature [66], increased metabolic rate [67], improved fitness and favorable body composition changes, and central nervous system fatigue [68]. The relationships between MVPA and mental and sleep health outcomes might be explained by physiological, psychological and social processes [69]. From a physiological perspective, engaging in MVPA may play a role in regulating the hormone response to stress induced by lockdown and a state of pandemic [70]. Furthermore, involvement in MVPA can provide social benefits through increasing time spent outdoors as well as the frequency and quality of social interactions and interpersonal relationships [71,72,73].

On the other hand, research on the associations between physical activity and sleep in the context of the pandemic and during lock-down periods is limited. Nevertheless, our findings are consistent with some emerging evidence demonstrating that physical activity outcomes, especially walking, are negatively impacted during periods of COVID-19 induced lockdown [37,74,75,76]. In other studies, physical activity levels remained unchanged or even improved, particularly among those with adequate physical activity levels prior to the lockdown suggesting that pre-pandemic physical activity levels may moderate these relations [13,37,77]. Similarly, studies investigating the impact of the pandemic on sleep have produced mixed results with some revealing increased prevalence of sleep problems and poor sleep quality [76,78], while others demonstrated improvements in sleep duration and quality [79]. However, there is a dearth in evidence on the nuanced relation between PA and sleep health during lockdown periods. While some data suggest that positive behavioral changes tended to cluster [77], others show that lockdowns may have reduced PA levels but improved some aspects of sleep health [37]. More research is necessary to disentangle these complex relations.

## 5. Recommendations for Future Research

Future research should continue to place emphasis on studying Middle Eastern populations, a population heavily understudied in public health research. There is a strong need for studies utilizing a large cohort and longitudinal studies with objective measures of PA and sleep. This allows researchers and policymakers to better understand the prevalence of poor sleep and physical inactivity in these populations and the complex pathway(s) linking physical activity, mental health, and sleep health to better inform public health policies and interventions in the region. This is particularly relevant during the global pandemic, as these behavioral and mental health factors influence immunity, chronic disease risk, and suicidality [80,81]. During periods of lockdown, it is important to consider intervention measures could prevent the deterioration of these health factors and their downstream effects on health span and disease risk, particularly in countries with limited healthcare resources. This includes the encouragement of transmission-safe activities, such as socially distanced outdoor solitary activity (e.g., walking and jogging). Further, the role of possible moderating factors such as gender, life stage, pre-pandemic physical activity levels, fitness levels warrant further consideration. Additionally, it is important to consider other interventions that directly improve mental and sleep health outcomes, including supporting people with mental health difficulties to enable them to engage in physical activity, in cases where poor mental health may be having a limiting influence on a person’s motivation/ability to exercise. Finally, future research should consider qualitative studies. Such studies can provide valuable insights into the mechanism(s) of association between physical activity and various health outcomes, and further elucidate the associations to better guide intervention measures and improve the health of the population in lockdown contexts.

## 6. Strengths and Limitations

This study had multiple noteworthy strengths. Firstly, this is among the first studies conducted on Middle Eastern populations to explore the impact of health behaviors, during periods of lockdown, that are risk factors for most prevalent chronic diseases. Such behaviors have been also linked to immunity and suicidality, which are both significant public health concerns during pandemics. Additionally, The Middle East continues to be heavily understudied in public health research and constitutes a region with limited public health data and limited access to resources to combat the pandemic relative to Western nations. Hence, our study is of particular importance to add essential public health data in the literature regarding the Middle Eastern population. Finally, this study utilized validated and widely used tools to measure physical activity, anxiety symptoms, depressive symptoms, and sleep health.

Despite these strengths, this study had limitations. Firstly, this study was cross-sectional in its nature. While it provides insightful information regarding associations between the exposure and outcome of interest, temporality cannot be established, especially for these likely bidirectional relations, and causal inference cannot be drawn. Furthermore, the self-reported survey is prone to social-desirability and recall bias. However, the online survey was the best possible method that could be used to collect data from a large sample during the COVID-19 induced lockdown in terms of the safety of participants and researchers. Although this study controlled for sociodemographic variables (i.e., age, gender, employment status, region of residence) in the analyses, other confounders such as the socioeconomic position, the size of household and crowding, neighborhood factors (e.g., walkability and noise levels), and the availability and accessibility of space and equipment for physical activity might have influenced the findings. Therefore, the possibility of residual confounding cannot be ruled out. Furthermore, it is important to note that the decision of categorizing MVPA as a dichotomous variable (e.g., meeting the MVPA guidelines or not) aids in the avoidance of forcing a functional form of the statistical relationship with our health outcomes. However, not using MVPA as a continuous variable may have introduced some limitations, as it may have allowed for elucidating further details about the relationship with other health outcomes. Finally, generalizability concerns are present given that the study sample is recruited from Facebook. Older adults, as well as individuals in lower socioeconomic statuses, may have been less likely to be represented among the sample.

## 7. Conclusions

Sufficient MVPA was associated with less depressive and anxiety symptoms, as well as better quality, longer duration, and less disturbance of sleep during a period of COVID-19 induced nation-wide lockdown in Jordan. Promoting physical activity during the lockdown could potentially improve mental and sleep health outcomes among the population. Longitudinal population-based studies with validated and objective measurement tools are needed to disentangle these complex associations and provide stronger evidence for guiding public health policies and interventions.

## Figures and Tables

**Table 1:** Participants’ characteristics (N = 1240).

Variable	Number (%)
Sociodemographic characteristics	
Age (years)	
18–24	127 (10.2)
25–29	188 (15.2)
30–39	471 (38.0)
40–49	271 (21.9)
≥50	172 (13.9)
Missing	11 (0.9)
Gender	
Male	656 (52.9)
Female	583 (47.0)
Missing	1 (0.1)
Employment status	
Employed	673 (54.3)
Self-employed	205 (16.5)
Student	84 (6.8)
Unemployed/retired	275 (22.2)
Missing	3 (0.2)
Region of residence ^a^	
Northern	237 (19.1)
Central	970 (78.2)
Southern	33 (2.7)
Physical activity	
Yes (≥150 min of MVPA per week)	391 (31.5)
No (<150 min of MVPA per week)	849 (68.5)
Mental health outcomes	
Anxiety	
Minimal/mild	1002 (80.8)
Moderate/severe	238 (19.2)
Depressive symptoms	
≤6	641 (51.7)
>6	599 (48.3)
Sleep health outcomes	
Sleep quality	
High	1018 (82.1)
Low	222 (17.9)
Sleep duration	
Sufficient (≥7 h)	660 (53.2)
Short (<7 h)	580 (46.8)
Sleep problems	
At least one problem	790 (63.7)
None	450 (36.3)

^a^ Northern region includes Irbid (N = 110), Jerash (N = 10), Balqa’a (N = 3), Zarqa (N = 91), Ajloun (N = 7), Mafrqa (N = 16); Central Region includes Amman (N = 925), Madaba (N = 19), Salt (N = 26); the southern region includes Tafila (N = 2), Karak (N = 6), Ma’an (N = 6), Aqaba (N = 15), and Petra (N = 4). MVPA = moderate-to-vigorous physical activity.

**Table 2:** Poisson regression models to demonstrate the associations between moderate-to-vigorous physical activity (MVPA) and mental and sleep health outcomes (N = 1240).

Variable	Measurement Scale	MVPA
		No (<150 min/week) ^a^
		aPR (95% CI) ^b^
Anxiety symptoms	Minimal/mild	Ref
	Moderate/severe	1.36 (1.05, 1.77) *
Depressive symptoms	≤6	Ref
	>6	1.30 (1.14, 1.49) **
Sleep quality	High	Ref
	Low	1.68 (1.24, 2.26) **
Sleep duration	Long (≥7 h)	Ref
	Short (<7 h)	1.15 (1.00, 1.31) *
Sleep problems	None	Ref
	At least one problems	1.22 (1.10, 1.35) **

^a^ Reference category = Yes (≥150 min/week; N = 391). ^b^ Adjusted for age, gender, region of residence and employment status. aPR = adjusted prevalence ratio; CI = confidence interval; Ref = reference. * *p* < 0.05; ** *p* < 0.01.

## Data Availability

The data presented in this study are available on request from the corresponding author. The data are not publicly available due to ongoing analyses and longitudinal study design purposes for future studies by the group.
